# Early experience with a composite ovine forestomach matrix graft in chronic lower extremity wounds: a multi-center retrospective case series

**DOI:** 10.1093/jscr/rjag129

**Published:** 2026-03-07

**Authors:** James E Geiger, Anthony J LaLama, Alpash K Patel

**Affiliations:** Northwestern Medical Group, Orland Park, IL, United States; Henry Ford Providence Southfield Hospital, Southfield, MI, United States; HCA Houston Healthcare, Sugar Land, TX, United States

**Keywords:** ovine forestomach matrix, hyaluronic acid, chronic wounds, lower extremity wounds

## Abstract

Chronic wounds place a significant burden on patients and healthcare. A newer outpatient treatment is a composite bioscaffold that contains ovine forestomach matrix and hyaluronic acid (OFM-HA). This multi-center retrospective case series investigated OFM-HA in the treatment of lower extremity chronic wounds. Medical records of 10 patients were reviewed and patient and wound characteristics were documented. Time to 50% percent area reduction (%PAR), time to closure, and complications were evaluated. Patients were primarily elderly with several comorbidities. The mean time to 50% PAR was 3.2 ± 2.3 weeks, and the mean time to closure was 9.9 ± 5.1 weeks. Patients received a median of 4 (IQR: 2, 5) applications. No complications or recurrences occurred. OFM-HA was effective and safe to treat complex lower extremity chronic wounds in the outpatient setting. Moreover, OFM-HA proved to be a durable material, with full closure requiring few applications.

## Introduction

Chronic lower extremity wounds present significant clinical, social and economic challenges that are expected to increase with rising rates of diabetes, obesity, and aging [1]. Chronic wounds are defined as soft tissue defects that do not resolve within 4 weeks of standard treatment [2–4]. Chronicity arises when healing fails to progress through normal phases, resulting in wounds that remain stalled in the inflammatory phase, marked by persistent pro-inflammatory cells and proteins, as well as excessive protease activity [4, 5].

Typical lower limb chronic wounds include diabetic foot ulcers (DFUs), pressure injuries (PIs), and venous leg ulcers (VLUs) [6]. However, wound clinics additionally manage atypical wounds like calciphylaxis and vasculitic ulcers [6]. Generally, standard treatment involves infection control, debridement, pressure relief, specialized dressings, compression wraps or pumps, and/or vascular surgery [6]. When these fail, advanced treatments like skin grafting, flap reconstruction, and skin substitutes (known as cellular, acellular, and matrix-like products, CAMPs) are used [6].

Ovine forestomach matrix (OFM) is a third-generation bioscaffold that has been commercialized as a range of products for both outpatient and inpatient care, with successful application in chronic wounds [7]. Preclinical data indicates that OFM contains a variety of proteins, including protease inhibitors, and enhances vascular density, supporting progression of stalled wounds [8, 9]. In a prospective cohort, OFM grafts promoted closure of Wagner 3 and 4 DFUs with no infections or graft loss, and a median of 1 OFM application [10]. Real-world, outpatient studies report faster DFU and VLU closure with OFM compared to reconstituted collagen [11, 12].

A composite OFM bioscaffold incorporating hyaluronic acid (OFM-HA) has been recently developed for the outpatient management of complex wounds [13]. OFM and hyaluronic acid (HA) synergistically promote keratinocyte proliferation and migration to help drive wound closure, while HA maintains a balanced moisture environment [13, 14]. Previous reports show successful treatment of chronic DFUs and calciphylaxis with OFM-HA [15, 16]. To expand on earlier findings, we retrospectively analysed outcomes from three outpatient wound care centers using OFM-HA to treat chronic wounds.

## Materials and methods

### General

All patients provided written informed consent for use of images and data. The study followed the Declaration of Helsinki ethical guidelines. Descriptive statistics were computed using GraphPad Prism (version 10.6.1). Normality was assessed using the Shapiro-Wilk test.

### Data collection

Retrospective data were collected from 10 patients with lower extremity chronic wounds treated with OFM-HA (Symphony™, Aroa Biosurgery Limited, Auckland, NZ) at three outpatient facilities between June 2022 and June 2025. The wounds were considered chronic if persistent for ≥4 weeks. Demographic information, baseline wound characteristics, wound area, and time to closure were recorded. Closure was defined as 100% re-epithelialization without drainage. Percent area reduction (%PAR) was calculated relative to the initial wound area.

### OFM-HA application

Before outpatient OFM-HA application, patients received standard of care (SOC), which included debridement, offloading, and secondary dressings; moreover, wounds were deemed infection-free, sharply debrided of non-viable tissue, and measured. OFM-HA devices were trimmed, hydrated (sterile saline), and secured with adhesive strips or staples. Wounds were dressed with a non-adherent dressing, followed by gauze and/or foam dressing, and appropriately compressed and/or offloaded. Patients were evaluated weekly, with wounds being cleansed and debrided and OFM-HA re-applied based on wound progression.

## Results

All wounds were chronic lower extremity ulcers treated with OFM-HA. The mean patient age was 67.7 ± 10.7 years and 70% were male. The median wound age was 4.5 (IQR: 4, 7.25) weeks with a median area of 6.8 (IQR: 3.8, 15.6) cm^2^ (Table 1). Most wounds were DFUs (70%), followed by a PI (10%), an atypical vasculitic ulcer (10%), and a necrotizing soft tissue infection (NSTI) resulting from a DFU (10%) (Table 1). Wound locations included the forefoot (70%), the heel (20%), and ankle (10%). Comorbidities included diabetes (90%), peripheral artery disease (80%), and hypertension (80%) (Table 1). Most patients had complicating factors, including prior amputations (40%) (Table 1). Three (30%) individuals had exposed bone.

**Table 1:** Patient demographics and wounds at baseline

Patient ID	Age/gender	Defect etiology/location	Comorbidities	Complicating factors	Previous treatments	Type of exposed structure	Defect age (weeks)	Initial wound area (cm^2^)	Initial max depth (cm)
1	74/M	DFU/forefoot	DM, PAD, CAD, HTN	Pacemaker, previous amputation, agent orange exposure neuropathy	SOC	bone	4.0	25.6	0.6
2	62/M	Pressure Injury/heel	DM, PAD, HIV, bladder cancer, Charcot arthropathy	N/A	SOC, IV antibiotics, 20 HBO dives	bone	6.0	25.0	1.5
3	65/M	DFU/forefoot	DM, PAD, HTN	N/A	N/A	bone	4.0	7.5	0.2
4	56/F	DFU/heel	DM, HTN, Charcot arthropathy	N/A	SOC	N/A	21.0	9.0	0
5	76/M	DFU/forefoot	DM, HLD, HTN	Previous amputation	SOC	N/A	4.0	3.0	0.2
6	73/F	DFU/forefoot	DM, HTN, PAD, HLD, CVA	Previous amputation	SOC, IV antibiotics	N/A	5.0	6.0	0.2
7	55/F	Atypical (vasculitic ulcers)/ankle	PAD, CVA, HTN, HLD, PVD, hypothyroid, autoimmune	Blindness	SOC, IV antibiotics	N/A	8.0	12.5	0.2
8	54/M	DFU, NSTI/forefoot	DM, PAD, HTN, PVD	N/A	N/A	N/A	4.0	4.5	0.2
9	79/M	DFU/forefoot	DM, PAD, HTN	Previous TMA	SOC, IV antibiotics	N/A	7.0	4.0	0.2
10	83/M	DFU/forefoot	DM, PAD, HTN, CKD, CAD	Previous amputation	SOC, IV antibiotics	N/A	4.0	1.28	0.1
**Mean ± SD**	68 ± 11						6.3 ± 5.6	9.8 ± 8.8	0.3 ± 0.4
**Median (IQR)**	69 (56, 77)						4.5 (4, 7.25)	6.8 (3.8, 15.6)	0.2 (0.2, 0.3)
**Min, Max**	54, 83						0, 21.0	1.3, 25.6	0, 1.5

Abbreviations: M = male, F = female; DFU = diabetic foot ulcer; HIV = human immunodeficiency virus; NSTI = necrotizing soft tissue infection; DM = diabetes mellitus; PAD = peripheral artery disease; CAD = coronary artery disease; HTN = hypertension; HLD = hyperlipidaemia; CVA = cerebrovascular accident; PVD = peripheral venous disease; CKD = chronic kidney disease; TMA = thrombotic microangiopathy; SOC = standard of care; HBO = hyperbaric oxygen therapy; IV = intravenous; SD = standard deviation; IQR = interquartile range; Min = minimum; Max = maximum; N/A = not applicable.

Wounds achieved a 50% PAR at a mean of 3.2 ± 2.3 weeks, and a mean time to closure of 9.9 ± 5.1 weeks (Table 2). Wounds received a median of 4 (IQR: 2, 5) OFM-HA applications (Table 2). The OFM-HA application rate (total applications/total weeks of OFM-HA treatment) was, on average, 0.4 ± 0.3 applications/week (Table 2). Notably, no complications or recurrences occurred in any of the patients (Table 2). The average time to the last follow-up was 36.2 ± 26.8 weeks, with one patient (#1, Table 2) lost to follow-up at week 8 and one patient (#2, Table 2) who was not seen after wound closure.

**Table 2:** Outcomes

Patient ID	Week achieved 50% PAR	Time to closure (weeks)	Number of applications	OFM-HA application rate (applications/week)	Time to last follow-up (weeks)	Complications
1	8.0	N/A (LTFU)	N/A (LTFU)	N/A (LTFU)	8.0	None
2	4.0	17.0	5	0.3	17.0	None
3	4.0	9.0	5	0.6	82.0	None
4	2.0	7.0	1	0.1	65.0	None
5	1.0	8.0	4	0.5	34.0	None
6	4.0	16.0	5	0.3	30.0	None
7	5.0	15.0	5	0.3	58.0	None
8	2.0	4.0	1	0.3	52.0	None
9	1.0	3.0	2	1.0	4.0	None
10	1.0	10.0	3	0.4	12.0	None
**Mean ± SD**	3.2 ± 2.3	9.9 ± 5.1	3 ± 2	0.4 ± 0.3	36.2 ± 26.8	
**Median (IQR)**	3.0 (1.0, 4.3)	9.0 (5.5, 15.5)	4 (2, 5)	0.3 (0.3, 0.6)	32 (11.0, 59.8)	
**Min, Max**	1.0, 8.0	3.0, 17.0	1, 5	0.1, 1.0	4.0, 82.0	

Abbreviations: SD = standard deviation; IQR = interquartile range; Min = minimum; Max = maximum; PAR = percent area reduction; N/A = not applicable; LTFU = lost to follow-up.

### Case example 1

**Figure 1:**
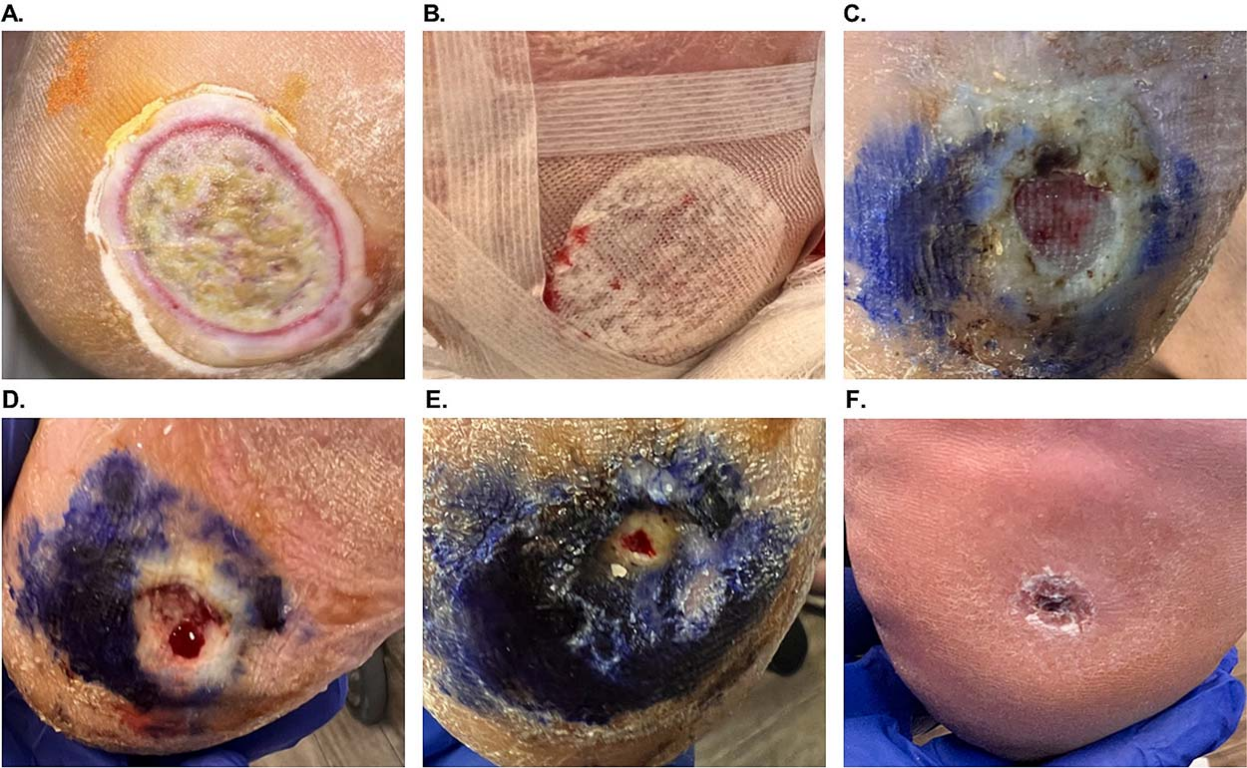
Patient #4 (left plantar heel Wagner 2 DFU) healing progression. (A) Initial presentation. (B) OFM-HA application and dressing. (C) Week 2 post-OFM-HA application, with fully integrated OFM-HA and reduced wound size. (D) Week 3 post-OFM-HA application and second sharp debridement. (E) Week 6 post-OFM-HA application and third sharp debridement, with further significant reduction in wound size. (F) Long-term follow-up, 5 months post-OFM-HA application.

A 56-year-old female (#4, Table 1) with uncontrolled diabetes, Charcot arthropathy, and hypertension presented with a 3 × 3 cm Wagner 2 DFU on the left plantar heel that persisted for 21 weeks and failed conservative care (Fig. 1A). After sharp debridement and hypochlorous acid irrigation, OFM-HA (5 × 5 cm) was applied (Fig. 1B). OFM-HA was fully integrated by week 2 and the wound had decreased by ~89% (wound size: 1.0 × 1.0 cm) (Fig. 1C). Following weekly visits, including a sharp debridement at week 3 (Fig. 1D), further reduction was observed at week 6 (wound size: 0.1 × 0.1 cm) (Fig. 1E). Complete closure was confirmed at 7 weeks after a single application of OFM-HA (not pictured). At 5 months, good tissue pliability and pigmentation was noted (Fig. 1F). No complications were reported.

### Case example 2

**Figure 2:**
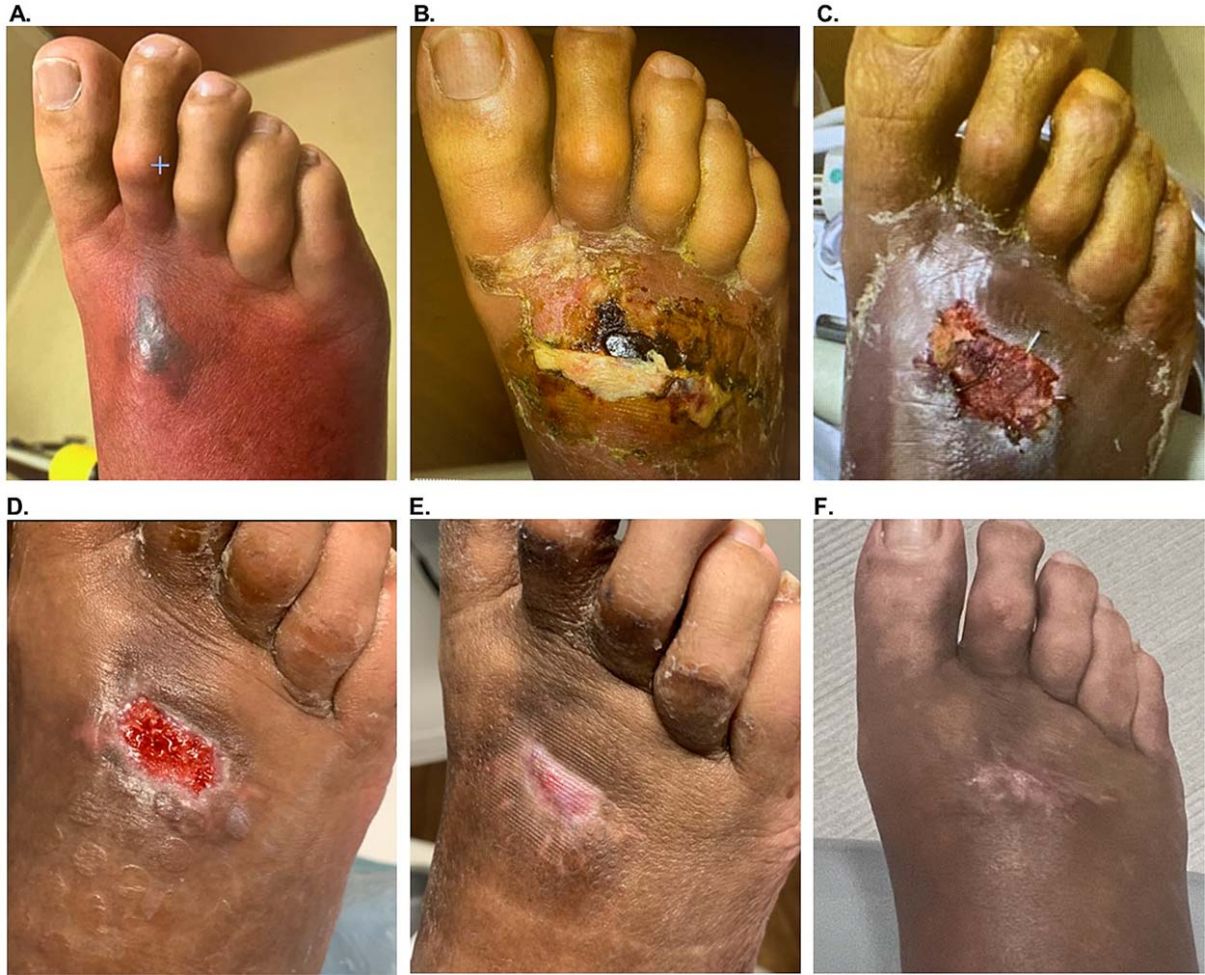
Patient #8 (right foot Wagner 3 DFU/NSTI) healing progression. (A). Initial presentation. (B) After incision and drainage, day of 3-layer OFM and OFM particulate application. (C) 1 week after application, with OFM matrix and particulate starting to integrate. (D) 4 weeks after OFM matrix and particulate application, with a fully vascularized wound bed, day of OFM-HA application. (E) 4 weeks post-OFM-HA application, with full closure. (F) Long-term follow-up, 11 weeks post-OFM-HA application.

A 54-year-old male (#8, Table 1) with uncontrolled diabetes, peripheral venous disease, peripheral artery disease, and hypertension presented with a Wagner 3 DFU with a concurrent NSTI on the dorsal side of the right foot (Fig. 2A). Intravenous antibiotics were prescribed to control the infection and reduce cellulitis, followed by incision and drainage, which resulted in a wound size of 6 × 4.5 × 0.2 cm (Fig. 2B). Subsequently, aggressive surgical debridement was followed by the application of OFM particulate (500 mg, Myriad Morcells™, Aroa Biosurgery Limited, Auckland, NZ) hydrated *in situ* with exudate and saline, as well as a 3-layer OFM graft (7 × 10 cm, Myriad Matrix™, Aroa Biosurgery Limited, Auckland, NZ) to fill wound depth and provide coverage over exposed structures. The OFM graft was well integrated one-week post-application (Fig. 2C), and completely integrated by 4 weeks post-operatively, producing a fully vascularized wound bed (Fig. 2D, wound size: 2 × 1.5 cm). To aid epithelial closure, OFM-HA (2.5 × 2.5 cm) was applied. At 4 weeks post-OFM-HA application, the wound fully closed (Fig. 2E). At the 11-week follow-up (Fig. 2F), and again at 1 year, the wound remained closed with normal pigmentation and pliability, as well as no complications or recurrences.

### Case example 3

**Figure 3:**
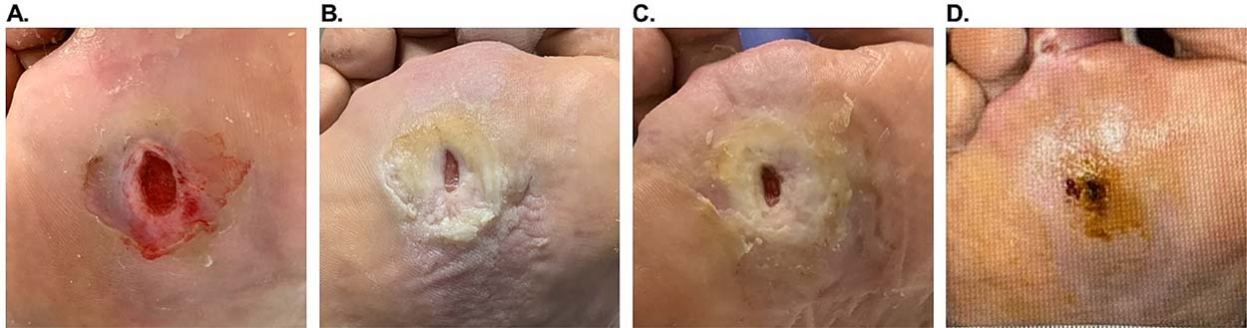
Patient #10 (right plantar region Wagner 2 DFU) healing progression. (A). Initial presentation, after debridement. (B). 1 week after the first OFM-HA application. (C) 4 weeks after the first OFM-HA application, day of the third application. (D) 10 weeks after the first OFM-HA application, full closure.

An 83-year-old male (#10, Table 1) with diabetes, peripheral artery disease, chronic kidney disease, coronary artery disease, hypertension, and a prior toe amputation presented with a Wagner 2 DFU on the right plantar region. After intravenous antibiotic administration and unsuccessful SOC, OFM-HA (2.5 × 2.5 cm) was applied after debridement (Fig. 3A, wound size: 1.6 × 0.8 × 0.1 cm). The wound area reduced by >50% after 1 week (Fig. 3B), with integration of OFM-HA. After maceration resolved, sharp debridement and repeat OFM-HA applications (16 mm disk) were performed at weeks 2 (not shown) and 4 (Fig. 3C, wound size: 0.6 × 0.4 cm). Notably, wound healing stalled between 2 and 8 weeks due to poor offloading. At 8 weeks, a single-layer OFM (Endoform Natural™, Aroa Biosurgery Limited, Auckland, NZ) was placed. Full closure occurred by week 10 with no complications (Fig. 3D), confirmed at week 12.

## Discussion

Lower extremity chronic wounds cause substantial morbidity and are difficult to resolve. When SOC fails, CAMPs, like OFM-HA, are increasingly used [15, 16]. While various outpatient CAMPs exist, OFM-HA is newer with limited clinical data. In this study, OFM-HA was effective in treating lower extremity chronic wounds in a particularly challenging patient group. Indeed, all patients responded well to OFM-HA, with 90% achieving a 50% reduction in wound area by 4 weeks, a 60% incidence of healing at 12-weeks, and a median time to healing of 9.0 weeks.

Of note, wounds received only ~1 application of OFM-HA every 2 weeks, with a median of 4 applications per patient, indicating the durability of OFM-HA in the chronic wound environment. In contrast, many outpatient CAMPs often require more frequent applications [17]. For example, a clinical study focused on amnion-based bioscaffolds reports up to 11 product applications [18]. Moreover, a similar fish xenograft study documents up to 16 applications per participant [19].

While successful closure of chronic wounds is challenging, patients in this case series achieved full closure in ~9.0 weeks. Additionally, no complications or recurrences were reported, consistent with prior published outcomes for OFM-HA [15, 16]. By comparison, some amnion-based product investigations report patients who never healed [20–22]. Furthermore, some outpatient fish skin-based xenograft studies report longer closure times, ranging from 10 to 17 weeks [19, 23].

The OFM technology has been commercialized as a range of devices, including OFM-HA, for both inpatient surgical reconstruction and outpatient complex wounds. For example, as a ‘collagen dressing,’ OFM (Endoform Natural™) is often utilized early in wound management as part of wound bed preparation [24, 25], or to aid epithelialization. OFM dressing provides an advanced technology for patients or care settings where access to CAMPs would otherwise be restricted by insurance coverage or cost. For complex surgical reconstruction, OFM has been fabricated into multi-layered grafts (Myriad Matrix™) and a morselized format (Myriad Morcells™). These products are used in the surgical setting, typically applied once with the aim of regenerating tissue over exposed structures or for volumetric fill. As we have shown in some of the cases presented herein, OFM-based products can be used together across the continuum of treatment, tailored to clinical objectives (e.g. wound bed preparation, epithelialization or tissue coverage) and importantly, financial considerations. For example, two patients (#3, #8) received an OFM graft and/or morselized OFM as part of a prior inpatient surgical reconstruction to achieve tissue coverage and fill, prior to wound closure with OFM-HA. This approach allowed the wounds to progress sufficiently in the inpatient setting prior to outpatient wound closure. In another patient (#10), the wound underwent three applications of OFM-HA over the course of 8 weeks, but healing progress was stalled due to lack of offloading compliance. To maintain continuity of care, the investigator elected to continue treatment with weekly applications of OFM dressing to facilitate final wound closure.

The current study has all the limitations of a retrospective case series, including a relatively small sample size, lack of randomization and a comparator cohort. Nonetheless, the study demonstrated that OFM-HA was effective in achieving wound closure in chronic lower extremity wounds. Further, no complications or recurrences were reported, from the relatively complex cohort. Future randomized studies are needed to validate these findings and make broader treatment recommendations regarding OFM-HA usage.
